# Improvement of DOPA-Melanin Production by *Aspergillus nidulans* Using Eco-Friendly and Inexpensive Substrates

**DOI:** 10.3390/jof9070714

**Published:** 2023-06-29

**Authors:** Beatriz Silva Campanhol, Beatriz Dias Ribeiro, Fernando Casellato, Kelly Johana Dussán Medina, Sandra Regina Pombeiro Sponchiado

**Affiliations:** 1 Department of Biochemistry and Organic Chemistry, Institute of Chemistry, São Paulo State University (UNESP), Araraquara 14800-060, SP, Brazil; bs.campanhol@unesp.br (B.S.C.); dias.ribeiro@unesp.br (B.D.R.); fernando.casellato@unesp.br (F.C.); 2 Department of Engineering, Physics and Mathematics, Institute of Chemistry, Sao Paulo State University (UNESP), Araraquara 14800-060, SP, Brazil; kelly.medina@unesp.br; 3 Bioenergy Research Institute (IPBEN), São Paulo State University (UNESP), Araraquara 14800-060, SP, Brazil

**Keywords:** optimization, pigment, corn steep liquor, molasses, vinasse, melanin, *Aspergillus nidulans*

## Abstract

Fungal pigments, including melanin, are recognized as promising materials for biomedical, environmental, and technological applications. In previous studies, we have demonstrated that the DOPA-melanin produced by the MEL1 mutant of *Aspergillus nidulans* exhibits antioxidant, anti-inflammatory, and antimicrobial activities without any cytotoxic or mutagenic effects, suggesting its potential use in pharmaceuticals. In order to increase the yield of this pigment and reduce the costs of its large-scale production, the present study aimed to evaluate agro-industrial by-products, sugarcane molasses, vinasse, and corn steep liquor as inexpensive substrates for fungal growth using experimental design methodology. According to the results obtained, the optimal composition of the culture medium was 0.81% (*v*/*v*) vinasse and 1.62% (*w*/*v*) glucose, which promoted a greater production of melanin (225.39 ± 4.52 mg g^−1^ of biomass), representing a 2.25-fold increase compared with the condition before optimization (100.32 mg.g^−1^ of biomass). Considering the amount of biomass obtained in the optimized condition, it was possible to obtain a total melanin production of 1 g L^−1^. Therefore, this formulation of a less complex and low-cost culture medium composition makes the large-scale process economically viable for future biotechnological applications of melanin produced by *A. nidulans.*

## 1. Introduction

The demand for pigments from natural sources is growing rapidly owing to increasing interest of the pharmaceutical, cosmetic, textile, and food industries to replace synthetic dyes with safer products that are easily degradable, eco-friendly, and do not have harmful effects on health human or the environment [1,2]. Production of microbial pigments is considered more advantageous than the extraction of pigments from plants and animals because microorganisms are considered microbial cell factories due to their ability to produce large amounts of compounds combined with the ease of large-scale cultivation using low-cost substrates [2,3,4,5,6].

Melanin, a natural pigment widely distributed in bacteria, fungi, algae, and other microorganisms, has attracted attention as an eco-friendly biomaterial with great potential for applications in biomedicine, dermocosmetics, nanotechnology, and bioengineering [7,8,9]. Studies have demonstrated that microbial melanin possesses a broad range of biological activities, including photo- and radio-protector, antitumor, antiviral, antioxidant, antimicrobial, neuroprotector, anti-inflammatory, immunomodulatory, antivenin, and metal chelator activities [2,7,9,10,11,12,13]. Owing to its various functional properties, high biocompatibility, and biodegradability; this pigment has been used in drug delivery systems, sunscreen and anti-aging formulations, anti-melanoma therapy, biosensors, tissue repair engineering, food packaging materials, metallic nanoparticles, optical biomimetics, and organic electronic devices [7,8,9,11,13,14,15,16,17].

Currently, commercially available melanin is obtained from *Sepia* ink and chemical synthesis, which makes its industrial application unfeasible. However, several species of fungi are able to produce different types of melanin, which are located in the outermost layer of the cell wall associated with chitin and/or outside the fungal cell, usually in the form of granules or as insoluble polymers in culture [18,19,20,21].

In a previous study, a mutant of the *Aspergillus nidulans* fungus was isolated and denominated MEL1; it presented an overproduction of DOPA-melanin [22]. This pigment exhibited antioxidant activity and was able to neutralize the radicals generated by hypochlorous acid [23]. It also showed anti-inflammatory activity, acting as an inhibitor of NO and TNF-α production in macrophages stimulated by bacterial lipopolysaccharide [24]. In addition, we also demonstrated that this pigment did not present cytotoxic effects in McCoy cell culture, even after metabolism by liver S9 fraction enzymes, and it had no mutagenic activity for the *Salmonella typhimurium* strains used in the Ames test [25]. These results suggest that the melanin produced by the MEL1 mutant has potential use in cosmetic formulations to protect the skin against possible oxidative damage and for the development of new therapeutic agents. In order to scale up the production of this pigment for future practical applications, it is necessary to determine the optimal cultivation conditions to increase the process efficiency. The composition of the medium is a critical factor for the economic feasibility of the process because it can represent about 38 to 73% of the total cost of production [4,9,11].

The utilization of the agro-industrial by-products as alternative substrates has been shown to be a promising strategy for cost-effective large-scale production due to its availability in large quantities with low commercial value and a composition rich in nutrients that could be used as sources of carbon, nitrogen, and minerals for microbial growth. Besides the economic advantages, their utilization prevents their disposal in the environment, which allows an eco-friendly production process [4,11,26,27].

Several studies have reported that the utilization of agro-industrial by-products ensures the cost-effective production of microbial pigments [4,9,28,29,30,31,32,33]. Molelekoa et al. [33] demonstrated the feasibility of the production of orange, green, yellow, red, and brown pigments by five filamentous fungi (*Penicillium multicolour*, *P. canescens*, *P. herquei*, *Talaromyces verruculosus*, and *Fusarium solani*) using a mixture of green waste and whey. Zou and Tian [34] showed that the optimization of a fermentation medium containing wheat bran extract as the main source of nutrients increased melanin production in *Auricularia auricula* by 10% compared with the non-optimized condition. Vasanthabharathi et al. [35] reported a higher melanin yield when *Streptomyces* sp. was grown in a medium containing 1% starch, 0.2% soybean meal, and 1% sugarcane waste. Arikan et al. [36] demonstrated that pomegranate pulp derived from the food processing industry had a high potential for pigment production, including melanin, by *Aspergillus carbonarius*. The halophilic black yeast *Hortaea werneckii* showed greater melanin production using glucose as a carbon source, peptone as a nitrogen source, and rice bran as a cheap carbon source [37]. In a previous study, the use of corn steep liquor, sugarcane bagasse hydrolysate, and pretreated sugarcane molasses was evaluated for the growth and production of melanin by the MEL1 mutant of the *Aspergillus nidulans* [38]. The results showed that the growth of the fungi was favored in the presence of 1–2% of corn steep liquor, while the highest pigment production (12% increase) was obtained at a lower concentration of corn steep liquor (0.2%). On the basis of these results, a two-step process for melanin production was established in which the cell growth (first stage) was stimulated in a medium supplemented with 1% of corn steep liquor, followed by pigment production (second stage), in which the medium was supplemented with 0.2% of corn steep liquor [39]. Thus, further studies were carried out in order to optimize the parameters of pH, temperature, agitation speed, and spore and pre-inoculum concentrations in two-step cultivation. The following optimal conditions were established: 10^6^ conidia mL^−1^; 1% corn steep liquor in the first stage; 10% pre-inoculum; 0.2% corn steep liquor and 0.2% Tween in the second stage; pH 6.8, 29 °C, and 225 rpm. This resulted in a 4.3-fold increase in pigment production compared with the previous condition [40].

Considering the great biotechnological potential of the melanin produced by the MEL1 mutant from *A. nidulans*, the aim of the present study was to evaluate the use of sugarcane molasses, vinasse, and corn steep liquor as components of the culture medium to maximize the production of this pigment and reduce the process costs on a large scale. For this propose, a 2^5−1^ fractional factorial design was used to assess the influence of glucose, sodium nitrate, corn steep liquor, molasses, and vinasse (independent variables) on pigment production (dependent variable); then, a 2^3^ central composite design (CCD) was applied to determine the optimal concentrations of nutrients that promoted greater pigment production by the MEL1 mutant. According to the results obtained, the optimization of the medium culture promoted a greater production of melanin using glucose as a carbon source and vinasse as an inexpensive substrate.

## 2. Materials and Methods

### 2.1. Microorganism

The MEL1 mutant of *A. nidulans* chosen for this work presents increased production of DOPA-melanin (*melC1*) and auxotrophy for inositol (*inoB1*) [22]. This mutant belongs to the culture collection of the Filamentous Fungi Laboratory at the Department of Biochemistry and Organic Chemistry, Institute of Chemistry, São Paulo State University-UNESP in Araraquara, Brazil.

### 2.2. Corn Steep Liquor, Vinasse, and Sugarcane Molasses

Vinasse and molasses were kindly provided by the São Martinho sugar/ethanol mill (Pradópolis/SP-Brazil), and the corn steep liquor was provided by Ingredion^®^ (Balsa Nova/PR/Brazil). These by-products were obtained from the same industrial batch to avoid variation in experimental results due to differences in composition between batches. They were filtered to remove solids and sterilized separately in the autoclave at 121 °C for 20 min prior to use in the culture medium.

### 2.3. Fungal Cultivation

The MEL1 mutant was first grown in a solid minimal medium, as described by Cove [41], supplemented with glucose 1% (*w*/*v*), sodium nitrate 0.6% (*w*/*v*), corn steep liquor 1% (*v*/*v*), and inositol 0.002% (*w*/*v*) for 5 days at 37 °C to obtain a conidial suspension.

The cultivation of the MEL1 mutant for melanin production was carried out in two steps, as established in previous studies by Sousa [40] and Sponchiado et al. [42]. In the first stage (preculture step), cultivation was carried out in Erlenmeyer flasks (500 mL) containing 200 mL of liquid minimal media, as described by Cove [41], supplemented with glucose 1% (*w*/*v*), sodium nitrate 0.6% (*w*/*v*), corn steep liquor 1% (*v*/*v*), and inositol 0.002% (*w*/*v*) and inoculated with 10^6^ spores mL^−1^ and kept at 29 °C in a rotatory shaker (225 rpm) for 48 h to promote fungal growth.

In the second stage (culture step), 10% (*v*/*v*) of the mycelial mass produced in preculture step was transferred to Erlenmeyer flasks (500 mL) containing 200 mL of liquid minimal media, as described by Cove [41], supplemented with glucose, sodium nitrate, corn steep liquor, molasses, and vinasse at different concentrations according to the design of experiments (DOE), and then they were incubated at 29 °C for 72 h in a rotatory shaker (225 rpm). After this period, the biomass was harvested by filtration, washed with deionized water, dried at 55 °C until reaching a constant weight, and crushed to obtain particles with diameters between 0.21 and 0.42 mm for the subsequent extraction of the pigment.

### 2.4. Optimization of MEL1 Mutant Culture Conditions for Melanin Production

In order to evaluate the influence of glucose, sodium nitrate, corn steep liquor, molasses, and vinasse (independent variables) on melanin production (dependent variable) during the culture step, a 2^5−1^ fractional factorial design was developed with a total of 19 runs with 3 replicates at the center point. Each independent variable was studied at three different levels, low (−1), intermediate (0), and high (+1), as shown in Table 1. The concentrations of corn steep liquor, molasses, and vinasse used in this design were chosen on the basis of previous studies [38,40].

Using the results of the fractional factorial design (2^5−1^), the significant variables that positively affected the production of melanin were analyzed and optimized by a 2^3^ central composite design (CCD). This experimental design consisted of 17 runs with 3 replicates at the center point, and five different levels (−1.68, −1, 0, + 1, and +1.68) of each independent variable were studied, as shown in Table 2. From the experimental results of CCD, a multiple regression analysis was applied, and a second-order polynomial equation was generated.

The response surface curves were constructed from the quadratic model in order to demonstrate the individual and interactive effects of statistically significant variables and determine the optimum concentrations of these variables for maximum melanin production by the MEL1 mutant.

### 2.5. Melanin Extraction

The melanin present in the mycelial mass was extracted according to the procedure described by Sava et al. [43] with modifications made by Gonçalves et al. [22]. The dried and ground biomass obtained after the fungus cultivation was transferred to a flask to which sodium hydroxide (NaOH) 1 mol L^−1^ was added at a proportion of 1:30 (*w*/*v*), followed by autoclaving at 121 °C for 10 min and centrifugation at 1935× *g* for 20 min at 20 °C. This procedure was repeated several times until complete depigmentation of the biomass was achieved. The supernatant containing the pigment was acidified with concentrated hydrochloric acid (HCl) until reaching pH 2 and kept at room temperature for 5 days. The precipitate obtained after centrifugation at 48,384× *g* for 20 min at 10 °C was washed 5 times in distilled water, dried at 60 °C for 24 h, and weighed. The amount of extracted melanin (not purified) was expressed in mg g^−1^ of biomass.

### 2.6. Melanin Purification and Quantification

Melanin purification was performed as described by Sava et al. [43] with minor modifications. The extracted pigment was hydrolyzed with 7 mol L^−1^ hydrochloric acid (HCl) at 100 °C for 2 h and subjected to centrifugation at 12,096× *g* for 15 min at 10 °C. The precipitate was washed with distilled water and then treated with organic solvents (chloroform, ethyl acetate, and ethanol). The sediment obtained after centrifugation at 1935× *g* for 15 min at 20 °C was dried at environmental room temperature and dissolved in 1 mol L^−1^ sodium hydroxide (NaOH), precipitated with 3 mol L^−1^ hydrochloric acid (HCl), and centrifuged at 12,096× *g* for 15 min at 10 °C. After these procedures, the purified melanin extract was washed with distilled water, dried at 60 °C, and weighed.

For quantification of purified melanin, following the protocol of Bull [44], the dry extract was re-dissolved in 0.5 mol NaOH (final concentration of 1 mg mL^−1^); the amount of melanin was determined by a spectrophotometer (Beckman Du^®^530) at 540 nm from a standard curve of synthetic DOPA-melanin (Sigma-Aldrich, purity ≥ 97%) and expressed in mg g^−1^ of biomass.

### 2.7. Statistical Analysis

The results obtained from the experimental designs were analyzed and interpreted using Statistica v10.0 software. Analysis of variance (ANOVA) was applied to determine the statistical significance of each factor on the response variable. The fit of the model was checked by the coefficient of determination (*R*^2^), and the statistical significance of the model equation was evaluated by Fischer’s test at a 5% level of significance.

## 3. Results and Discussion

Initially, a 2^5−1^ fractional factorial design was used (Table 3) to evaluate the effects of glucose, sodium nitrate, molasses, vinasse, and corn steep liquor on melanin production by the MEL1 mutant. This strategy also allowed the selection of the statistically significant variables that promoted an increase in the production of this pigment.

By analyzing the results obtained in the 2^5−1^ fractional design (Table 3), it is possible to notice a great variation in the amount of melanin produced, ranging from 12.23 to 192.64 mg g^−1^ of biomass, indicating that the agro-industrial by-products present in the fungal culture significantly affected the production of this pigment by the MEL1 mutant. These results are in agreement with those of other studies, which have shown that nutritional and environmental factors, including the composition of the culture medium, the type and concentration of carbon and nitrogen sources, temperature, light intensity, aeration, agitation, and pH, influence cell growth and, consequently, the production of fungal pigments [3,30].

From the data presented in Table 3, the main effects of each independent variable were estimated, which showed that all tested variables were statistically significant (*p* < 0.10) for melanin production (Table 4 and Figure S1). Molasses, vinasse, and glucose showed significant effects at higher levels, which means that melanin production increased as the concentration of these components increased. This analysis confirmed the results obtained from the 2^5−1^ fractional factorial design (Table 3), in which the highest melanin production was obtained in the culture medium containing molasses, vinasse, and glucose (run 7). On the other hand, the corn steep liquor and sodium nitrate had significant effects at their lowest levels, i.e., an increase in the production of this pigment was obtained by decreasing their concentration in the culture medium (run 7). Then, the variables with significant effects at their higher levels, molasses, vinasse, and glucose were set to 2% (*v*/*v*), 1% (*v*/*v*), and 1% (*w*/*v*) respectively, while those with significant effects at their lower levels (corn steep liquor and sodium nitrate) were not considered for further optimization because the lowest level of these factors was equal to 0% (*v*/*v*).

On the basis of the analysis of the significant effects obtained from the 2^5−1^ fractional design, a 2^3^ Central Composite Design (CCD) was employed to evaluate the influence of glucose, vinasse, and molasses (significant variables) and determine their optimal concentrations in order to maximize melanin production by the MEL1 mutant.

According to CCD results (Table 5), there was an increase in the amount of pigment produced, but with a smaller variation (from 129.85 to 218.21 mg g^−1^ of biomass) compared with the previous design (Table 3). These results showed that the selection of independent variables and their concentrations were correctly applied, demonstrating their importance for the production of melanin by the MEL1 mutant. The values obtained at the central points (runs 15–17) also presented little variation, indicating good reproducibility of the experimental data.

**Figure 1:**
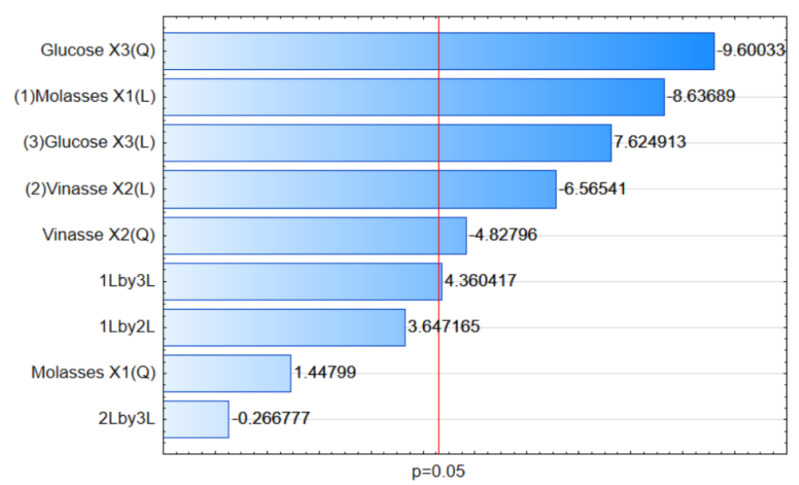
Pareto chart for the effects of independent variables and their interactions with melanin production by the MEL1 mutant, according to the 2^3^ CCD.

In order to assess the effects of each selected variable (molasses, vinasse, and glucose) as well as the interaction between them in the production of melanin (response variable), a Pareto chart was constructed (Figure 1). As can be observed, all variables (in linear terms) were statistically significant (*p* < 0.05), confirming their great influence on the melanin produced by the MEL1 mutant.

However, molasses and vinasse had significant effects at their lowest levels (Figure 1), indicating that an increase in the concentration of these variables caused a decrease in melanin yield. These results are in agreement with those presented in Table 5, where, in the presence of higher concentrations of molasses (2.45%) and vinasse (2.40%), there was a reduction in the production of this pigment (166.52 mg g^−1^ of biomass, run 12); in comparison, a greater amount of melanin was produced (218.21 and 202.85 mg g^−1^ of biomass, respectively, for runs 9 and 11) when molasses and vinasse were not present in the culture medium.

Conversely, glucose showed a significant effect at its highest level (Figure 1), which means that an increase in its concentration promoted greater melanin production. As shown in Table 5, a large amount of this pigment was produced (180.14 mg g^−1^ of biomass, run 14) at a high concentration of glucose (2.44%), while a lower yield was obtained (148.83 mg g^−1^ of biomass, run 13) when glucose was not present in the culture medium.

From the regression analysis of the experimental data, a second-order model was proposed to predict the melanin production as a function of the significant variables (*p* < 0.05) according to the following Equation (1):

(1) $$y=194.47-23.16x_{1}-17.62x_{2}-14.26x_{2}^{2}+20.46x_{3}-28.35x_{3}^{2}+15.29x_{1}x_{3}$$

where *y* is the response (melanin production) and $x_{1}$, $x_{2}$, $x_{3}$ are the real values of the independent variables (molasses, vinasse, and glucose, respectively).

The analysis of variance (ANOVA) results presented in Table 6 suggest that the proposed model is valid to predict the amount of melanin produced by the MEL1 mutant since the calculated F (4.64) was statistically significant compared with the critical *F* (3.44) with *p* < 0.05. The coefficient of determination obtained by the analysis (R^2^ = 0.8225) indicated that 82% of the variability in the observed response values could be explained by the model, confirming a satisfactory adjustment of the proposed model to the experimental data. In addition, the fit of the statistical model to the experimental data was confirmed by the lack-of-fit value for regression, which was not significant (*p* > 0.05).

**Figure 2:**
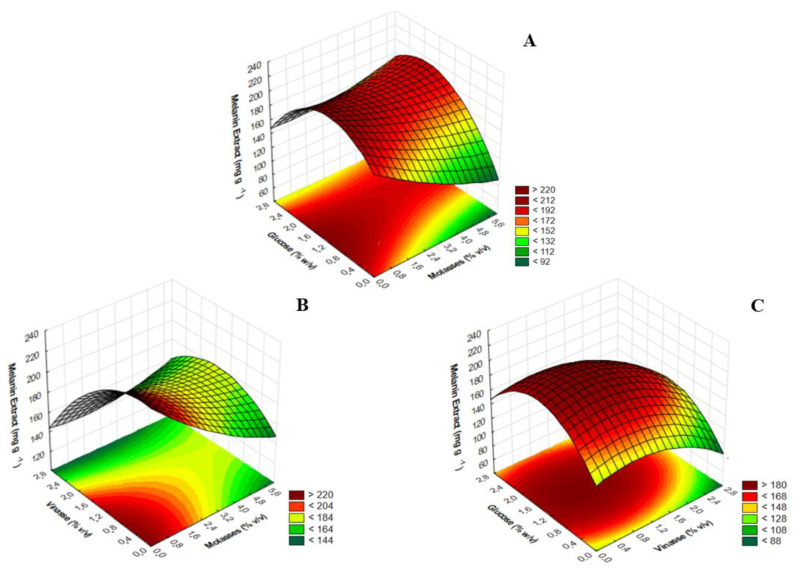
Response surface generated by the model showing the effect of interactions between glucose and molasses (**A**); molasses and vinasse (**B**); and vinasse and glucose (**C**) on melanin production by the MEL1 mutant.

The three-dimensional response surface (Figure 2) was generated on the basis of the proposed model to analyze the interaction between the selected variables and determine the optimal levels of each variable required for maximum melanin production by the MEL1 mutant.

By analyzing the glucose and molasses interaction (Figure 2A), it is possible to observe that greater production of melanin occurred at higher glucose concentrations, ranging from 1.2% to 1.6%, while molasses was at a concentration of zero. In the interaction between vinasse and molasses, shown in Figure 2B, increased production of this pigment was also achieved when vinasse was at a lower concentration (around 0.8%) and molasses was not present in the culture medium. The interaction between vinasse and glucose (Figure 2C) also showed that there was greater melanin production at a low vinasse concentration (around 0.8%), but glucose needed to be present at high concentrations, ranging from 1.2% to 1.6%. This analysis is in agreement with the results from the 2^3^ CCD (Table 5), which showed that the highest melanin production (218.21 mg g^−1^ of biomass) occurred in the culture medium containing only vinasse and glucose (run 9).

Our results confirmed that carbon sources, mainly glucose, played a vital role in fungal metabolism, providing energy for cellular activities as well as pigment biosynthesis [45]. The exclusion of molasses from the culture medium can be explained by the presence of certain components, such as heavy metals, which may not favor or even inhibit pigment synthesis [30]. The addition of vinasse at a low concentration may be due to the presence of phenols, which may favor the production of melanin, acting as a substrate for its synthesis; however, they can also be toxic at high concentrations [46]. Likewise, vinasse may contain copper, which acts as an activator of enzymes involved in the pathway of melanin synthesis but may exert an inhibitory effect at higher concentrations [47,48]. In a previous optimization study [49], a greater melanization of the MEL1 mutant was achieved in the presence of L-DOPA and L-tyrosine (melanin precursors) and copper sulfate; however, it was required at a very low concentration (0.27 µmol L^−1^).

In order to ensure the validation of the model proposed by the 2^3^ CCD, the MEL1 mutant was grown in the culture medium supplemented with the predicted optimal concentrations of each variable, estimated by the desirability function. The optimized formulation of the culture medium for greater pigment production was established with 0.81% (*v*/*v*) of vinasse and 1.62% (*w*/*v*) of glucose, which resulted in a pigment production equal to 291.74 mg g^−1^ of biomass. This value was higher than the one predicted by the model (223.11 mg g^−1^ of biomass), corresponding to a 1.3-fold increase, indicating a high degree of accuracy of the proposed model.

By analyzing melanin production before and after optimization (Table 7), it was possible to verify that there was an increase in the production of this pigment under the optimal conditions determined in this study. After pigment purification, the amount of pure melanin obtained in the optimized condition was 225.39 mg g^−1^ of biomass, which represented a 2.25-fold increase compared with the condition before optimization (100.32 mg g^−1^ of biomass). It is important to note that the total melanin production reached 1 g L^−1^, considering the amount of biomass produced in the optimized condition (Table 7). Comparing these results with others described in the literature, lower melanin yields have been obtained when only agro-industrial by-products have been used as substrates for the production of this pigment in fungi [7]. Zou and Tian [34] reported that, after the optimization of a medium containing wheat bran extract as the main source of nutrients, the highest melanin production by *Auricularia* was equal to 0.52 g L^−1^. Mujdeci [50] obtained 0.19 g L^−1^ intracellular melanin produced by *Aureobasidium pullulans* NBRC 100716 using carrot peel extracts as fermentation media. Therefore, in the present study, the optimization of the culture medium resulted in a very high production of melanin by the MEL1 mutant using vinasse as a low-cost substrate and glucose as a carbon source.

## 4. Conclusions

From optimization studies using fractional factorial design followed by central composite design, it was possible to select the significant variables and determine their optimal concentration to maximize melanin production by the MEL1 mutant. The composition of the optimized culture medium, containing 0.81% (*v*/*v*) of vinasse and 1.62% (*w*/*v*) of glucose, promoted a 2.25-fold increase in melanin production compared with the previous condition. Therefore, with the optimization of the medium culture, it was possible to obtain a total yield of melanin equal to 1 g L^−1^ using glucose as the carbon source and vinasse as an inexpensive substrate. This formulation of less complex culture media with the presence of the agro-industrial by-products represents a cost reduction in the large-scale production process, allowing future biotechnological applications of melanin produced by the MEL1 mutant of *A. nidulans.*

## Figures and Tables

**Table 1:** Concentrations of independent variables at different levels used in the 2^5−1^ fractional factorial design.

		Levels		
Variables	Coded Value	−1	0	+1
Corn steep liquor (% *v*/*v*)	X_1_	0	0.50	1.00
Molasses (% *v*/*v*)	X_2_	0	1.00	2.00
Vinasse (% *v*/*v*)	X_3_	0	0.50	1.00
Sodium nitrate (% *w*/*v*)	X_4_	0	0.30	0.60
Glucose (% *w*/*v*)	X_5_	0	0.50	1.00

**Table 2:** Concentrations of independent variables at different levels used in the 2^3^ central composite design (CCD).

		Levels				
Variables	Coded Value	−1.68	−1	0	+1	+1.68
Molasses (% *v*/*v*)	X_1_	0.01	1.00	2.45	3.90	4.89
Vinasse (% *v*/*v*)	X_2_	0.01	0.50	1.23	1.95	2.44
Glucose (% *w*/*v*)	X_3_	0.01	0.50	1.23	1.95	2.44

**Table 3:** Matrix of 2^5−1^ fractional factorial design for the effect of independent variables (with real and coded values) on the melanin production by the MEL1 mutant.

Run	Corn Steep Liquor (*v*/*v*)	Molasses (*v*/*v*)	Vinasse (*v*/*v*)	Sodium Nitrate (*w*/*v*)	Glucose (*w*/*v*)	Melanin Extract ^(a)^ (mg g^−1^ of Biomass)
**1**	−1 (0%)	−1 (0%)	−1 (0%)	−1 (0%)	1 (1%)	173.26
**2**	1 (1%)	−1 (0%)	−1 (0%)	−1 (0%)	−1 (0%)	22.86
**3**	−1 (0%)	1 (2%)	−1 (0%)	−1 (0%)	−1 (0%)	133.33
**4**	1 (1%)	1 (2%)	−1 (0%)	−1 (0%)	1 (1%)	151.04
**5**	−1 (0%)	−1 (0%)	1 (1%)	−1 (0%)	−1 (0%)	32.97
**6**	1 (1%)	−1 (0%)	1 (1%)	−1 (0%)	1 (1%)	46.46
**7**	−1 (0%)	1 (2%)	1 (1%)	−1 (0%)	1 (1%)	192.64
**8**	1 (1%)	1 (2%)	1 (1%)	−1 (0%)	−1 (0%)	74.98
**9**	−1 (0%)	−1 (0%)	−1 (0%)	1 (0.6%)	−1 (0%)	25.97
**10**	1 (1%)	−1 (0%)	−1 (0%)	1 (0.6%)	1 (1%)	53.43
**11**	−1 (0%)	1 (2%)	−1 (0%)	1 (0.6%)	1 (1%)	158.21
**12**	1 (1%)	1 (2%)	−1 (0%)	1 (0.6%)	−1 (0%)	57.08
**13**	−1 (0%)	−1 (0%)	1 (1%)	1 (0.6%)	1 (1%)	181.22
**14**	1 (1%)	−1 (0%)	1 (1%)	1 (0.6%)	−1 (0%)	12.23
**15**	−1 (0%)	1 (2%)	1 (1%)	1 (0.6%)	−1 (0%)	174.39
**16**	1 (1%)	1 (2%)	1 (1%)	1 (0.6%)	1 (1%)	99.75
**17**	0 (0.5%)	0 (1%)	0 (0.5%)	0 (0.3%)	0 (0.5%)	96.52
**18**	0 (0.5%)	0 (1%)	0 (0.5%)	0 (0.3%)	0 (0.5%)	94.90
**19**	0 (0.5%)	0 (1%)	0 (0.5%)	0 (0.3%)	0 (0.5%)	90.52

^(a)^ Melanin Extract refers to unpurified melanin.

**Table 4:** Estimated effects of independent variables on the melanin production by the MEL1 mutant from the 2^5−1^ fractional factorial design.

Factors	Coded Value	Effect	Pure Error	t (2)	*p* ^(a)^
Mean		98.51	0.71	138.34	0.000052
Corn steep liquor (% *v*/*v*)	X_1_	−69.27	1.55	−44.63	0.000502
Molasses (% *v*/*v*)	X_2_	61.63	1.55	39.71	0.000634
Vinasse (% *v*/*v*)	X_3_	4.93	1.55	3.18	0.086373
Sodium Nitrate (% *w*/*v*)	X_4_	−8.16	1.55	−5.26	0.034350
Glucose (% *w*/*v*)	X_5_	65.27	1.55	42.06	0.000565

^(a)^ All factors are statistically significant at 90% confidence level (*p* < 0.10).

**Table 5:** Matrix of 2^3^ central composite design for the effect of independent variables (with real and coded values) on melanin production by the MEL1 mutant.

Runs	Molasses (*v*/*v*)	Vinasse (*v*/*v*)	Glucose (*w*/*v*)	Melanin Extract ^(a)^ (mg g^−1^ of Biomass)
1	−1 (1.00%)	−1 (0.50%)	−1 (0.50%)	197.92
2	1 (3.90%)	−1 (0.50%)	−1 (0.50%)	129.85
3	−1 (1.00%)	1 (1.95%)	−1 (0.50%)	164.59
4	1 (3.90%)	1 (1.95%)	−1 (0.50%)	135.58
5	−1 (1.00%)	−1 (0.50%)	1 (1.95%)	198.81
6	1 (3.90%)	−1 (0.50%)	1 (1.95%)	174.80
7	−1 (1.00%)	1(1.95%)	1(1.95%)	177.29
8	1 (3.90%)	1 (1.95%)	1 (1.95%)	165.11
9 *	−1.68 (0.00%)	0 (1.23%)	0 (1.23%)	218.21
10 *	1.68 (4.89%)	0 (1.23%)	0 (1.23%)	203.56
11 *	0 (2.45%)	−1.68 (0.00%)	0 (1.23%)	202.85
12 *	0 (2.45%)	1.68 (2.40%)	0 (1.23%)	166.52
13 *	0 (2.45%)	0 (1.23%)	−1.68 (0.00%)	148.83
14 *	0 (2.45%)	0 (1.23%)	1.68 (2.44%)	180.14
15 **	0 (2.45%)	0 (1.23%)	0 (1.23%)	189.74
16 **	0 (2.45%)	0 (1.23%)	0 (1.23%)	191.29
17 **	0 (2.45%)	0 (1.23%)	0 (1.23%)	199.00

^(a)^ Melanin Extract refers to unpurified melanin; * axial points; ** central points.

**Table 6:** Analysis of variance (ANOVA) of the second-order model for the melanin production by the MEL1 mutant from the 2^3^ CCD.

Variation Factor	Sum of Squares	Degrees of Freedom	Medium Square	F Calc.	F Crit.	*p*-Value
**Regression**	8157.928	8	1019.741	4.639118	3.44	0.021948 *
**Residual**	1758.508	8	219.8136			
**Lack of fit**	1709.302	6	284.8836	11.57904	19.33	0.081619
**Pure error**	49.20678	2	24.60339			
**Total**	9916.437	16				

(R^2^ = 0.8225). * Significant with *p* < 0.05.

**Table 7:** Production of biomass and melanin by the MEL1 mutant before and after optimization of the culture medium.

Conditions	Biomass (g L^−1^)	Purified Melanin (mg g^−1^ of Biomass)
Before optimization	3.92 ± 0.06	100.32 ± 28.13
After optimization	4.57 ± 0.16	225.39 ± 4.52

## Data Availability

Not applicable.

## Supplementary Materials

The following supporting information can be downloaded at: https://www.mdpi.com/article/10.3390/jof9070714/s1, Figure S1: Pareto chart for the effects of independent variables on melanin production by MEL1 mutant, according to the 2^5–1^ fractional factorial design.
