# Supplementary material for: Improvement of DOPA-Melanin Production by Aspergillus nidulans Using Eco-Friendly and Inexpensive Substrates
Source: J Fungi (Basel). 2023 Jun 29;9(7):714. doi: 10.3390/jof9070714 (PMC10381910; doi:10.3390/jof9070714)
Supplement: Supplementary file 1 [file jof-09-00714-s001.zip › jof-2368620-supplementary.pdf]

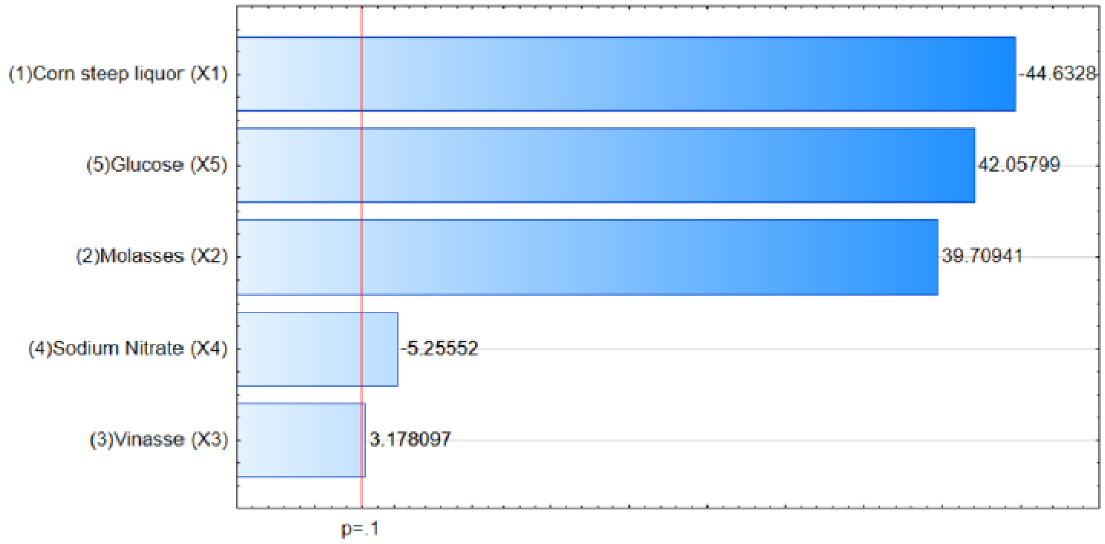

Figure S1. Pareto chart for the effects of independent variables on melanin production by MEL1 mutant, according to the 2^{5-1} fractional factorial design.
